# Prosthodontic Rehabilitation of the Flabby Edentulous Maxilla Using Hobkirk’s Technique: A Case Report

**DOI:** 10.7759/cureus.61292

**Published:** 2024-05-29

**Authors:** Rupal Lalwani, Sheetal R Khubchandani, Anjali Bhoyar, Surekha A Dubey

**Affiliations:** 1 Prosthodontics, Sharad Pawar Dental College & Hospital, Datta Meghe Institute of Medical Sciences, Wardha, IND

**Keywords:** polyvinyl siloxane, hobkirk’s technique, impression technique, flabby ridge, edentulism

## Abstract

Edentulism is characterized by the loss of teeth, which can significantly impact oral health and the quality of life of older patients. Among the challenges faced by individuals with edentulism is the occurrence of flabby ridges, a common consequence of prolonged tooth loss. Flabby ridges, characterized by soft, compressible tissue in the edentulous ridge area, present unique management challenges for dental professionals. Fibrous or flabby alveolar ridges present challenges in the fabrication of predictable prostheses. Impression making, a critical step in prosthodontic treatment, becomes particularly problematic as forces exerted during the process can distort the mobile denture-bearing tissues, which will cause the denture to become unstable and loose. This case report aims at an impression technique that can enhance the treatment outcomes for edentulous patients with fibrous alveolar ridges as this decreases the pressure over flabby tissue.

## Introduction

The quality of life can be significantly impacted by masticatory inefficiency. Hence, it is critical to ascertain how different prostheses affect mastication. The patient's general health and ability to masticate are impacted by tooth loss [1]. Flabby tissue refers to the excessive soft tissue found in an edentulous area mostly seen over the anterior maxillary ridge, and this condition frequently results in poor denture stability and retention. Studies have shown that fibrous ridges are existing in about 24% of edentate maxillae and 5% of the edentate mandible. Additionally, surgical ridge augmentation procedures are recommended as possible solutions for flabby ridges. On the other hand, surgically removing loose tissue raises the volume of the denture material and removes the cushion of soft connective tissues that absorb tension, which may injure the tissues underneath. Consequently, the treatment of flabby ridges is more frequently accomplished by the use of conventional prosthodontic treatments, such as balancing occlusal loads along with specific impression techniques [2].

The "impression making of the flabby ridge" involves taking an impression of this soft tissue area. During this process, the soft tissue is displaced by the impression material. However, the fibrous tissue underlying the flabby ridge tends to spring back or recoil to its original position once the impression is removed. This recoil action helps displace the final denture prosthesis, ensuring a more accurate fit and better retention of the denture.

In essence, this means that, while the impression process temporarily displaces the soft tissue, the inherent elasticity of the fibrous tissue allows it to return to its original position, which in turn influences the fit and placement of the final denture prosthesis [3]. Therefore, there is a need for recording the tissues in a static state, accordingly various techniques of impression have been developed. Thus, while fabricating dentures on flabby tissues, an assessment of the correct technique and material is required [4]. Hence, the following report presents a case about the management of flabby tissue using Hobkirk’s window technique.

## Case presentation

**Figure 1:**
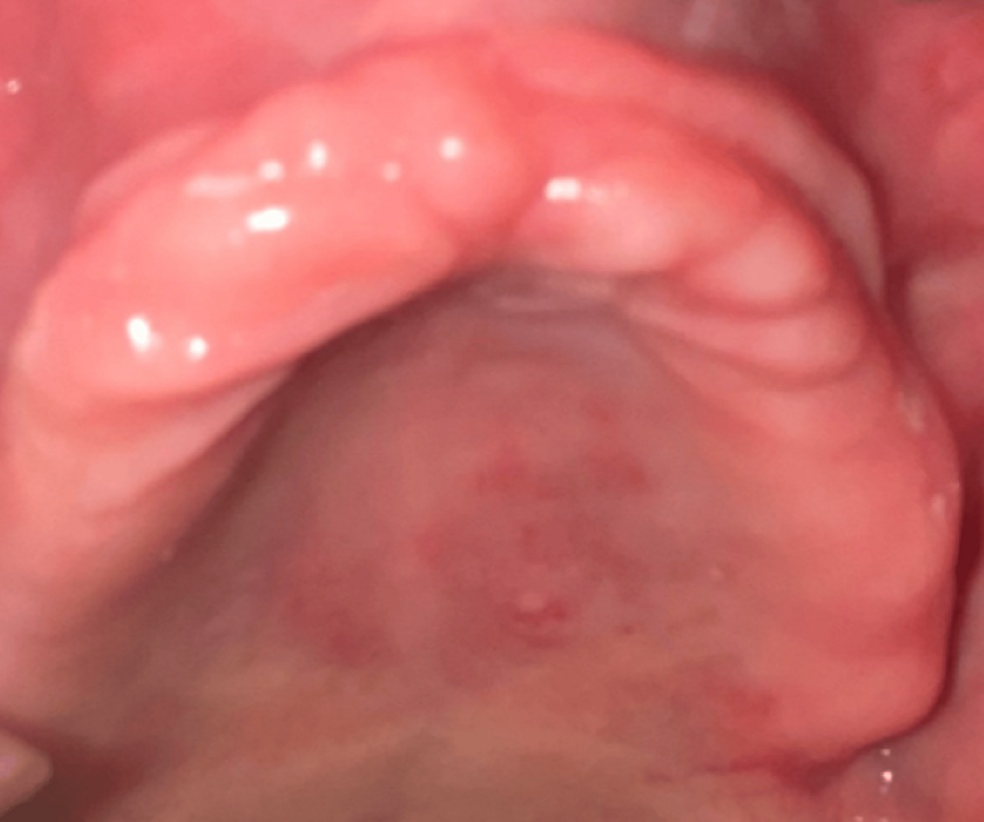
Flabby anterior maxillary edentulous ridge

A 64-year-old elderly woman patient reported to the Department of Prosthodontics, Sharad Pawar Dental College and Hospital, Sawangi Meghe, Wardha, India, with the chief complaints of missing teeth and masticatory inefficiency, and wanted to get them replaced with new complete dentures. A complete case history was recorded. Extra-oral and intra-oral examination was done. Intra-oral examination revealed fibrous tissue, which was present over the anterior maxillae region (Figure 1). Maxillary denture prosthesis was fabricated using a modified impression technique (i.e., Hobkirk's technique for the flabby ridge), and all the steps were followed conventionally.

**Figure 2:**
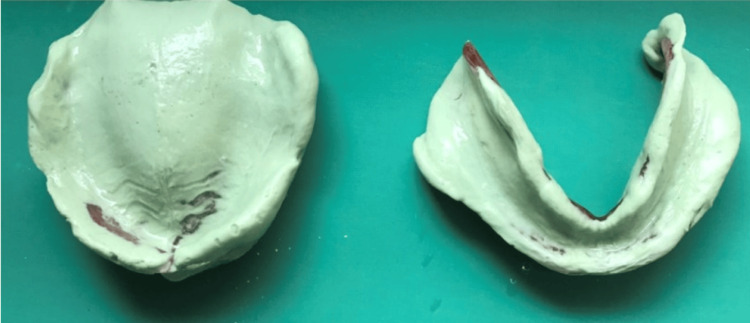
Preliminary impression of the edentulous ridge using irreversible hydrocolloid

**Figure 3:**
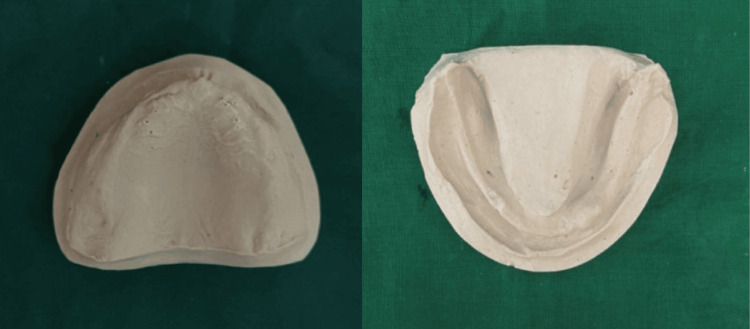
Primary cast

To reduce the movement of the fibrous tissue, an irreversible hydrocolloid material using a stock metal perforated tray for edentulous patients was used to record preliminary impressions (Figure 2). Impressions were poured using a type III gypsum product, and primary casts were obtained (Figure 3).

**Figure 4:**
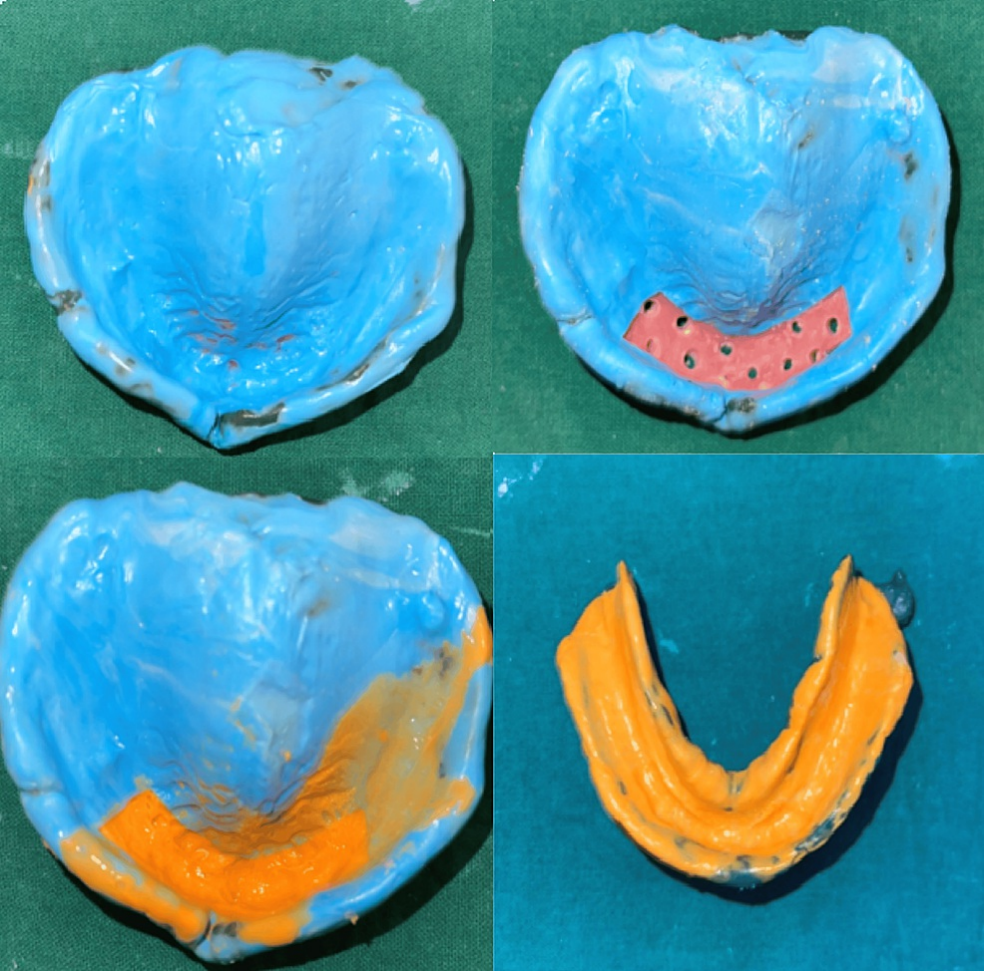
The final impression was made with Hobkirk’s technique using the polyvinyl siloxane elastomeric impression

**Figure 5:**
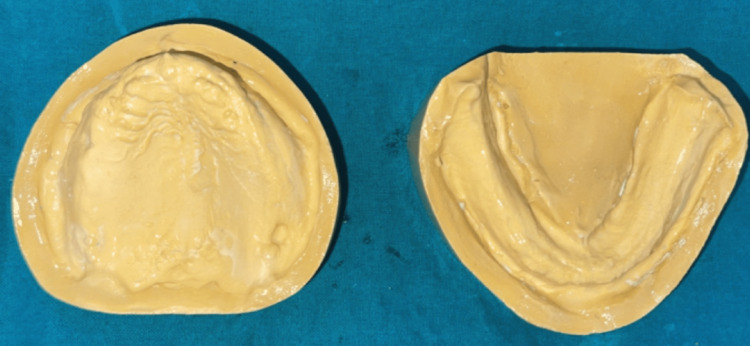
Final impression poured with dental stone

Auto-polymerizing cold cure acrylic resin was used to fabricate a special tray, and the tray's borders were shortened by 2 mm of the sulcular depth. Type II impression compound (i.e., green stick) was used for border molding. The anterior part of the specialized tray, which will coincide with anterior flabby ridge venting holes, was prepared using fissured and round bur. The final impression was made after removing spacer wax with polyvinyl siloxane (PVS) material. A light-body PVS material was first loaded in the syringe and then injected over the exposed site through the window to record flabby tissue in a static state (Figure 4). The master cast was prepared by pouring a definitive impression (Figure 5).

**Figure 6:**
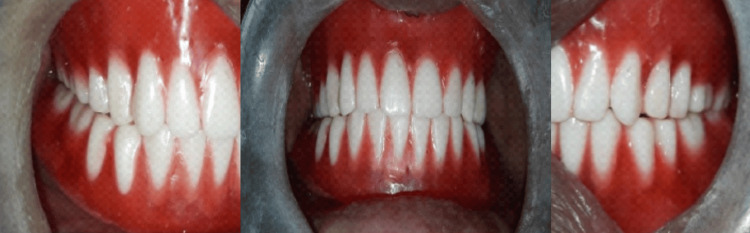
Try in of the denture

**Figure 7:**
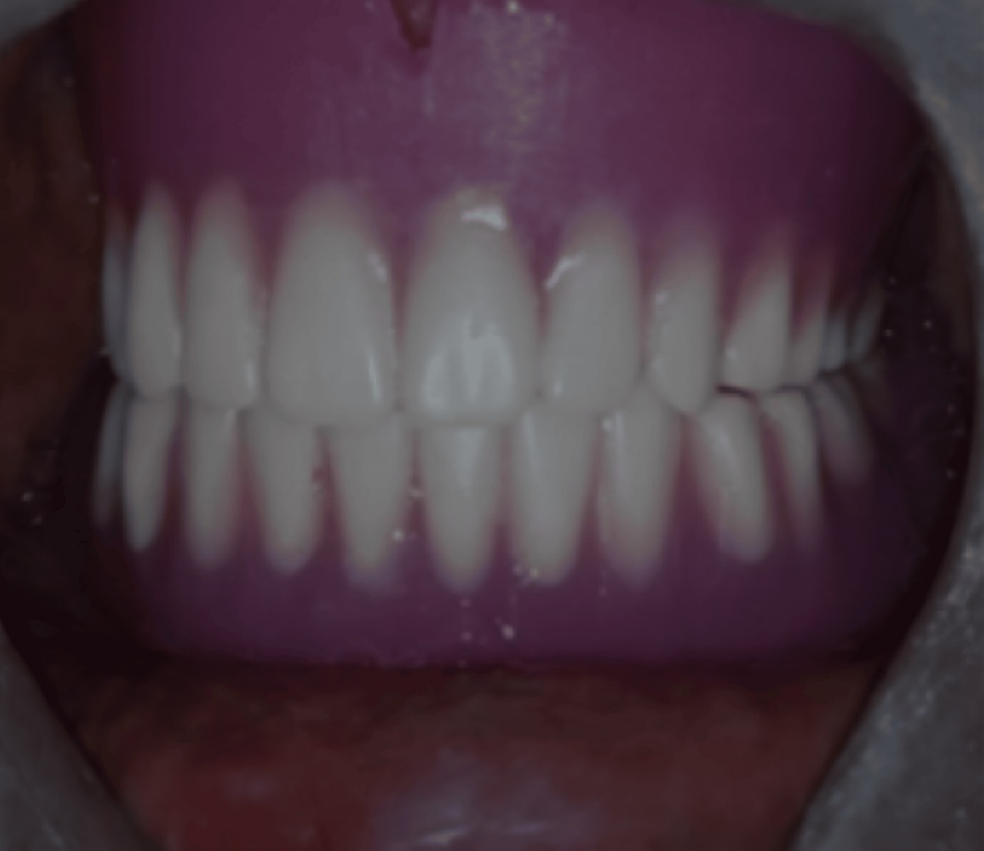
Final denture

**Figure 8:**
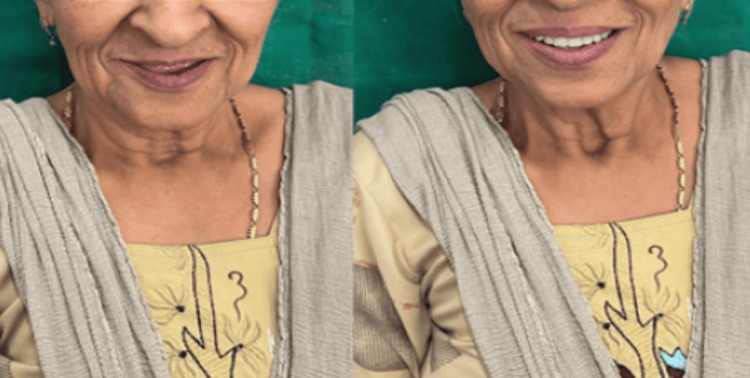
Before and after insertion of the denture

The rest all the laboratory and clinical steps of complete denture fabrication were performed according to the conventional technique. A try-in of dentures was done (Figure 6). Dentures were delivered to the patient, and post-insertion instructions were given (Figure 7). Follow-up was conducted at 24 hours, one week, one month, and six months (Figure 8).

## Discussion

Management of flabby tissues/ridges is a critical issue faced by many clinicians and prosthodontists particularly. Various non-surgical and surgical techniques can be advocated for the same. Surgically removing fibrous tissues and then restoring them with a prosthesis is a complex procedure. Implant-retained prosthesis enhances denture stability, but it is expensive and has time constraints. Such problems can be overcome by conventional prosthodontic treatment, with some technique adjustments [5].

In 1964, Liddlelow reported an approach that employed two distinct impression materials. A special tray was used. Plaster of Paris was used for the flabby ridge area and zinc oxide eugenol (ZnOE) for the healthy ridge. In 1964, Osborne additionally explained the recording of normal and flabby tissues independently using two different materials and trays and then comparing them intra-orally. In 1986, Watt and McGregor indicated a method that uses the impression compound to a customized tray and final impression with ZnOE impression paste [6,7]. These techniques have been used by many authors for decades and have been used nowadays also with some modifications. For example, Colvenkar et al. have used the same techniques in their cases with slight modifications.

Other commonly described techniques in the literature include Watson's window technique, Filler's two-tray technique, and Zafrulla Khan's Window technique [5].

With the palatal splinting technique, there is a possibility of minimal distortion, particularly anteriorly during the first stage, and compression of the ridge during the second impression stage [8]. A single-step impression procedure utilizing a medium-body elastomeric material is the Shum & Pow technique [4]. This approach is less time-consuming, offers patient comfort, eliminates the need for fabricating two custom trays, and results in minimal distortion of edentate arches compared to other materials [4].

Conventional impression techniques for recording flabby tissues often lead to the displacement of fibrous tissue, which subsequently returns to its anatomical position, causing displacement of the final denture prosthesis. Introducing holes or windows, or incorporating wax reliefs, reduces hydraulic pressure during the impression-making of flabby areas, minimizing tissue distortion and displacement [9].

Plaster of Paris material was substituted with a light-body PVS impression material to record the tissue in a static state. Plaster of Paris presents challenges in uniform application and controlling a low-viscosity material on flabby tissues due to patient positioning and gravity, leading to inconsistent results. PVS materials are also appropriate for mucostatic and muco-compressive impressions because offer various viscosities [10].

Furthermore, the open tray lets dental professionals see how well the impression material has adapted to the flabby tissue. Thus, in the fabrication of complete dentures, the authors suggested the clinical adoption of this modified window technique [11]. Every method has its own limitations and clinical challenges. The only limitation of the technique is that it is static and hence arbitrary windows are made.

## Conclusions

This case report details a straightforward method for rehabilitating an edentulous patient with a fibrous ridge, focusing on fabricating a denture using the window impression technique for the flabby tissue. In this method, a light-body PVS impression material was used to record the shape of the soft ridge without displacing it. This differs from using impression plaster, which can be poured through exposed windows but may require undercuts in the PVS material to ensure proper retention. Furthermore, there is no need for further clinical steps with this approach. This tactic enhanced denture-related results and enhanced the patient's overall health. This strategy enhanced denture-related outcomes and positively impacted the patient's overall well-being.
